# Dystrophin Dp71 Subisoforms Localize to the Mitochondria of Human Cells

**DOI:** 10.3390/life11090978

**Published:** 2021-09-16

**Authors:** Emma Tabe Eko Niba, Hiroyuki Awano, Tomoko Lee, Yasuhiro Takeshima, Masakazu Shinohara, Hisahide Nishio, Masafumi Matsuo

**Affiliations:** 1 Department of Community Medicine and Social Healthcare Science, Kobe University Graduate School of Medicine, Kobe 650-0017, Japan; mashino@med.kobe-u.ac.jp; 2 Department of Pediatrics, Kobe University Graduate School of Medicine, Kobe 650-0017, Japan; awahiro@med.kobe-u.ac.jp; 3 Department of Pediatrics, Hyogo College of Medicine, Nishinomiya 663-8501, Japan; to-ri@hyo-med.ac.jp (T.L.); ytake@hyo-med.ac.jp (Y.T.); 4 Department of Occupational Therapy, Faculty of Rehabilitation, Kobe Gakuin University, Kobe 651-2180, Japan; nishio@reha.kobrgakuin.ac.jp; 5 Department of Physical Therapy, Faculty of Rehabilitation, Kobe Gakuin University, Kobe 651-2180, Japan; mmatsuo@reha.kobegakuin.ac.jp

**Keywords:** Duchenne muscular dystrophy, dystrophin, mitochondria, Dp71 subisoforms

## Abstract

Duchenne muscular dystrophy (DMD) is a fatal muscle wasting disease caused by deficiency in dystrophin, a protein product encoded by the *DMD* gene. Mitochondrial dysfunction is now attracting much attention as a central player in DMD pathology. However, dystrophin has never been explored in human mitochondria. Here, we analyzed dystrophin in cDNAs and mitochondrial fractions of human cells. Mitochondrial fraction was obtained using a magnetic-associated cell sorting (MACS) technology. Dystrophin was analyzed by reverse transcription (RT)-PCR and western blotting using an antibody against the dystrophin C-terminal. In isolated mitochondrial fraction from HEK293 cells, dystrophin was revealed as a band corresponding to Dp71b and Dp71ab subisoforms. Additionally, in mitochondria from HeLa, SH-SY5Y, CCL-136 and HepG2 cells, signals for Dp71b and Dp71ab were revealed as well. Concomitantly, *dystrophin* mRNAs encoding *Dp71b* and *Dp71ab* were disclosed by RT-PCR in these cells. Primary cultured myocytes from three dystrophinopathy patients showed various levels of mitochondrial Dp71 expression. Coherently, levels of mRNA were different in all cells reflecting the protein content, which indicated predominant accumulation of *Dp71*. Dystrophin was demonstrated to be localized to human mitochondrial fraction, specifically as Dp71 subisoforms. Myocytes derived from dystrophinopathy patients manifested different levels of mitochondrial Dp71, with higher expression revealed in myocytes from Becker muscular dystrophy (BMD) patient-derived myocytes.

## 1. Introduction

Duchenne muscular dystrophy (DMD) (OMIM 310200) is one of the most common inherited muscular diseases, affecting more than one in every 3500 live-born male children and characterized by progressive muscle wasting, succumbing at the second to fourth decades of life [1]. DMD is caused by dystrophin deficiency due to mutations in the *DMD* gene [2]. The gene spans more than 2.4 Mb on chromosome X and encodes a 14-kb transcript comprising 79 exons [3]. Furthermore, the gene has been known to encode at least 7 alternative promoters/first exons that produce promoter- or tissue-specific dystrophin isoforms [3,4]. Another development-specific promoter/first exon was identified to produce a full-length transcript, Dystrophin protein (Dp) 412e, giving further complexity in transcript [5].

Dystrophin isoforms are named according to their corresponding molecular mass: the full-length and smallest ones are Dp427 [3] and Dp40 [6], respectively. Dp40 promoter/first exon is located in intron 62 spliced to exon 63 [7]. Dystrophin, actually Dp427m (muscle isoform), is localized beneath the skeletal muscle membrane and links the extracellular protein with intracellular actin by forming the dystrophin-dystroglycan complex [8,9]. It is now well-established that dystrophin plays an important role in the safeguarding of the subsarcolemmal cytoskeleton of striated muscles [10,11,12]. Its deficiency causes DMD [2,12]. Dystrophin is a multi-functional protein and besides its involvement with DMD, its absence also contributes to nervous system breakdown and exacerbation of energy metabolism [11].

Another dystrophin isoform expressed from the same promoter as Dp40 is a protein with approximately 71 kDa in molecular mass, Dp71 [13]. Dp71 is ubiquitously expressed in all tissues and has a role in many biological processes including cognitive impairment [14], retinal abnormality [15], cell adhesion and cell division [16], nuclear architecture formation [17] and water homeostasis [18].

Although the cardinal symptom of DMD is skeletal muscle wasting triggered by dystrophin deficiency, downstream events such as respiratory distress, cardiomyopathy and scoliosis resulting from muscle wasting have been well documented [19,20]. Before the identification of dystrophin as a responsible protein of DMD, mitochondria (MT) dysfunction had been suspected to be an important pathogenic feature in DMD [21]. However, studies on MT in DMD have not been very active after dystrophin identification.

Meanwhile, disturbances of MT metabolism in dystrophin-deficient mouse muscle tissue [22], exacerbation of DMD pathology by a complex I deficiency [23], and deregulation of calcium permeability in MT [20] have been reported. Consistently, Timpani et al. has proposed a hypothesis that DMD is primarily an MT myopathy in which the inability to generate sufficient quantities of ATP induces the pathophysiological cascade of events leading to muscle wasting [24]. Hence, MT impairment is central to disease etiology rather than a secondary pathophysiological consequence of dystrophin deficiency [24]. In the skeletal muscle of the *mdx* mouse, a DMD model mouse, dystrophin was demonstrated to be a requirement for the maintenance of the subsarcolemmal MT-pool density [25]. This implicated dystrophin as a player in the spatial control of MT localization.

The above results strongly suggested some dystrophin-MT interaction. However, dystrophin isoforms’ expression in MT has not been characterized to the best of our knowledge. One study, however, disclosed the localization of dystrophin into brain-derived MT from *mdx* mice [26]. From their findings, a subisoform of Dp71, Dp71f (lacking exon 78) was found to be expressed in the *mdx* mouse brain-derived MT. In addition, another subisoform Dp71d (harboring exon 78) was not detected in MT from *mdx* mouse brain [26]. Nonetheless, no study on dystrophin localization in the MT of human cells has been conducted so far.

HEK293 cells are cells established from human embryonic kidney tumors. In our previous study, Dp71 was shown to be expressed in HEK293 [27] as well as human satellite cells [28]. The Dp71 expressed in HEK293 cells comprised two subisoforms, Dp71b and Dp71ab [27]. Dp71b is 72.2 kDa while Dp71ab is a 70.8 kDa protein, respectively [29], both are encoded by the G-dystrophin transcript lacking exon 78. This exon deletion leads to the production of a group of isoforms termed Dp71f [30]. Dp71b was identified in both the cytoplasmic and nuclear fractions of HEK293, while Dp71ab was shown specifically localized to the nucleus [27]. With the Dp71 subisoform specific localization exhibited by this cell line, it is highly expected that this cell line could be amenable for the elucidation of the roles of human Dp71 isoform similarly, as mouse PC12 cells have been used to establish the roles of mouse Dp71 isoform [31]. This isolated subcellular expression of Dp71 subisoforms could represent a good model for exploration.

In this study, dystrophin in the MT fraction of HEK293 cells was analyzed by western blot assay and compared to other human cancer cell lines, as well as primary cultured myocytes from three dystrophinopathy patients. Subisoforms of dystrophin Dp71 were shown to localize to the MT fraction.

## 2. Materials and Methods

### 2.1. Cell Lines and Primary Cultured Myocytes

HEK293, HeLa, SH-SY5Y, CCL-136 and HT-29, KATOIII were obtained from ATCC (American type culture collection, Manassas, VA, USA) and the European Collection of Cell Cultures (ECACC, Salisbury, UK), respectively. HEK293, HeLa, HepG2 and CCL-136 cells were cultured in Dulbecco’s Modified Eagle’s Medium (DMEM), SH-SY5Y cells were cultured in DMEM / Ham’s F12 medium, HT-29 cells were cultured in McCOY’s 5A medium and KATOIII cells were cultured in IMDM medium. All media were purchased from Wako (Wako Pure Chemical Industries Ltd., Osaka, Japan) supplemented with 10% fetal bovine serum (FBS; Gibco by Life Technologies, Grand Island, NY, USA) except for KATOIII cells (20% FBS) and 1% Antibiotic-Antimycotic (Gibco by Thermofisher, Scientific Inc., Boston, MA, USA). Cell cultures were maintained at 37 °C in a 95% O_2_ and 5% CO_2_ humidified incubator.

Primary cultured myocytes were established from three dystrophinopathy patients (two DMD and one BMD). All procedures were performed with the informed consent and ethical clearance of the Kobe University Ethical Committee.

Myocytes were cultured in dishes coated with type I collagen (Sumilon, Osaka, Japan) in DMEM containing high glucose supplemented with 20% FBS, 1% antibiotics-antimycotics and 2% Ultroser-G (Biosepra, Cedex-Saint-Christophe, France) and maintained at 37 °C in a 95% O_2_ and 5% CO_2_ humidified incubator.

### 2.2. Isolation of MT Fraction

MT fraction of HEK293 and other cell lines were isolated using the MT Isolation Kit, human (Miltenyl Biotech, Bergisch Gladbach, Germany). As the protocol included in the kit was not proper for a less abundant protein like dystrophin, the procedure was amended as reported before [32]. Briefly, 3 × 10^6^ cells were suspended with 1 mL lysis buffer and homogenized with a 29G X 1/2” needle (BD Transduction Laboratories, Franklin Lakes, NJ, USA). The lysed cells were mixed with 9 mL of cold 1× separation buffer (SB) and 50 mL anti-TOM22 magnetic microbeads and incubated for 60 min at 4 °C. Meanwhile, the LS column was washed with 3 mL of SB. Next, the labelled lysate was applied to the LS column and separated by the help of a magnetic field. Then the anti-TOM22 magnetic microbeads were bound to the LS column. The column was washed three times with 3 mL SB, removed from the magnetic separator and placed in a clean 15 mL tube. SB buffer of 1.5 mL volume was added into the LS column and the MT was immediately flushed out of it with a plunger. The eluted MT pellet was collected by centrifugation at 13,000× *g* for 2 min and suspended in 50–100 mL storage buffer and stored at −80 °C in a freezer until required. Alternatively, the protocol was modified with two additional steps for the acquisition of non-cytosolic-contaminated pure MT: (1) a glass homogenizer was employed, and cells were confirmed to be at least 80% lysed by trypan blue. Then the lysed cells were centrifuged for 10 min at low speed of 1000× *g* and the supernatant was used for MT extraction as above, (2) After flushing out the MT fraction was mixed with 9 mL of cold 1× SB, reapplied to a new column and the isolation process repeated a second time. Total protein concentrations of the eluted MT were measured with a Qubit 4.0 Fluorometer (Invitrogen, Thermofisher Scientific Inc., Boston, MA, USA).

### 2.3. Isolation of Nuclear and Cytoplasmic Fractions

To investigate the localization of dystrophin in the nucleus and cytoplasmic fractions, HEK293 cell culture corresponding to 2 × 10^6^ cells were collected. Nuclear and cytoplasmic fractions were obtained using NE-PER (Pierce Biotechnology, Rockford, IL, USA) as previously reported [33]. An aliquot corresponding to approximately 40 μg of each fraction was used for protein analysis.

### 2.4. Protein Analysis by Western Blotting

Total cell protein lysates were prepared by homogenizing cells with the Radioimmunoprecipitation assay (RIPA) buffer and sonication. All total protein concentrations were measured with a Qubit 4.0 Fluorometer (Invitrogen, Thermofisher Scientific Inc., Boston, MA, USA).

An aliquot of cell fractions or total cell lysate corresponding to 40–80 μg was electrophoresed by 10% sodium dodecyl sulphate-polyacrylamide gel electrophoresis (SDS-PAGE), transferred to a polyvinylidene difluoride (PVDF) membrane and blotted using a semi-dry blotting system (Biorad laboratories Inc., Hercules, CA, USA). Blotting membranes were blocked with ECL reagents (GE life science, Little Chalfont, UK) in Tris-buffered saline with Tween-20 and subsequently reacted overnight with antibodies against rabbit anti-dystrophin (Ab15277), mouse anti-VDAC1/Porin (mitochondrial porin family; ab14734), mouse anti-COXIV (cytochrome C oxidase IV family; ab14744), and mouse anti-ATP5A (ATPase alpha/beta chains family; ab14748) purchased from abcam (Abcam, Cambridge, UK), and mouse anti-β-actin (sc-47778) and histone 4 (sc-8650) were purchased from Santa Cruz Biotechnology (Santa Cruz Biotechnology Inc., Santa Cruz, CA, USA). All primary antibodies were used at a dilution of 1:1000 except for β-actin, which was used at a dilution of 1:5000. After washing, membranes were reacted with a horseradish peroxidase-conjugated goat anti-mouse IgG (1:10,000; Promega, Fitchburg, MA, USA), donkey anti-goat IgG (1:10,000; sc-2020; Santa Cruz Biotechnology Inc., CA, USA) or goat anti-rabbit IgG antibody (1:10,000; Promega). Immunoreactivity was detected with an ImmobilonTM Western chemiluminescent HRP substrate (Millipore Corporation, Billerica, MA, USA) and visualized using a ChemiDoc XRS Molecular Imager (BioRad Laboratories Inc., Hercules, CA, USA). Volumetric analysis of each band was done by the inbuilt software (BioRad Laboratories Inc., Hercules, CA, USA).

### 2.5. RNA Isolation and RT-PCR

Cultured cells were rinsed twice with phosphate buffered saline (Sigma-Aldrich Co., St. Louis, MO, USA) and then collected using Lysis/Binding Buffer of High Pure RNA isolation kit (Roche Diagnostics, Basel, Switzerland). RNA was extracted according to the manufacturer’s instructions (Roche Diagnostics, Basel, Switzerland). Complementary DNA was synthesized from 0.5 µg of each total RNA using random primers as described previously [34]. PCR amplification was performed in a total volume of 10 µL, containing 1 µL of cDNA, 1 µL of 10× ExTaq buffer (Takara Bio, Inc., Shiga, Japan), 0.25 U of *ExTaq* polymerase (Takara Bio, Inc., Shiga, Japan), 500 nM of each primer and 250 µM dNTPs (Takara Bio, Inc., Shiga, Japan). Thirty cycles of amplification were performed on a Mastercycler Gradient PCR machine (Eppendorf, Hamburg, Germany) using the following conditions: initial denaturation at 94 °C for 5 min, subsequent denaturation at 94 °C for 1 min, annealing at 60 °C for 90 secs and extension at 72 °C for 1 min. The region spanning *DMD* exons 70 to 79 was PCR amplified using two primer pairs: c70; 5′-CAGGAGAAGATGTTCGAGAC-3′ as the forward primer and 5F; 5′-ATCATCTGCCATGTGGAAAAG-3′ as the reverse primer. The *dystrophin* transcript was amplified by reverse transcription (RT)- PCR as was the *glyceraldehyde dehydrogenase (GAPDH)* gene [35]. PCR-amplified products were electrophoresed using a DNA 12,000 LabChip kit on an Agilent 2100 Bioanalyzer (Agilent Technologies, Santa Clara, CA, USA). Peak heights were quantified with the inbuilt software (Agilent Technologies, Santa Clara, CA, USA).

### 2.6. DNA Sequencing

PCR-amplified products visualized by agarose gel electrophoresis were excised from the gel with a sharp razor blade, pooled, and purified using a QIAGEN gel extraction kit (QIAGEN, Inc., Hilden, Germany). Purified products were subcloned into the pT7 blue T vector (Novagen, Inc., San Diego, CA, USA) and submitted for direct sequencing. All sequencing analyses were conducted by Greiner Bio-One Co. Ltd. (Tokyo, Japan).

### 2.7. Immunoprecipitation

Immunoprecipitation was investigated using the Dynabeads^®^ Co-Immunoprecipitation (Co-IP) kit (Novex, Thermofisher, Scientific Inc., Boston, MA, USA). The mouse anti-ATP5A (2 μg; ab14748; Abcam, Cambridge, UK) or mouse anti-dystrophin (7A10) antibody (20 μg; sc-47760, Santa Cruz Biotechnology Inc., CA, USA) that detects an epitope corresponding to amino acids 3558–3684 of dystrophin was coupled with 10 mg of Dynabeads^®^ M-270 Epoxy overnight at 4 °C. The antibody coupled beads were washed and extracted in a magnetic field. 1.5 mg of antibody coupled beads was mixed with 1g of total protein lysate derived from DMD3-derived primary cultured myocytes for dynabeads Co-IP complex preparation as specified by the protocol. The protein lysate for this assay was prepared with lysis buffer containing 0.1% of SDS. The complexes were washed, and the purified protein was separated through a magnetic field and transferred to a clean tube. A fraction was mixed with sample buffer (BioRad Laboratories Inc., Hercules, CA, USA), boiled for 5 min at 95 °C and used for SDS-PAGE and western blot to probe for dystrophin and MT proteins. To investigate for non-specific binding, a normal rabbit IgG (sc-2027; Santa Cruz Biotechnology Inc., Santa Cruz, CA, USA) was also used as a western blotting control.

### 2.8. Statistical Analysis

All assays were carried out in triplicate and statistical analyses were performed using Microsoft Excel with the add-in software Statcel 3 (The Publisher OMS Ltd., Tokyo, Japan). Results reported as the means ± SD were analyzed by Student’s t-tests for comparisons between two groups. A * *p* < 0.05 and ** *p* < 0.01 was considered to be statistically significant.

## 3. Results

### 3.1. Isolation of MT Fraction from HEK293 Cells

The association of Dp71 with MT was examined in HEK293 human embryonal kidney cells. The initial isolation was performed following an already optimized protocol [32] and the isolated MT fraction was examined for the expression of dystrophin, β-actin (cytoplasmic marker) and COXIV (MT marker) by western blotting. From the result, using an antibody against the C-terminal region, dystrophin Dp71 could be visualized in the MT fraction as double bands. Nevertheless, both signals for β-actin and COXIV were also observed in the MT fraction (Data not shown). This suggested incomplete separation of MT from the cytoplasmic fraction.

**Figure 1:**
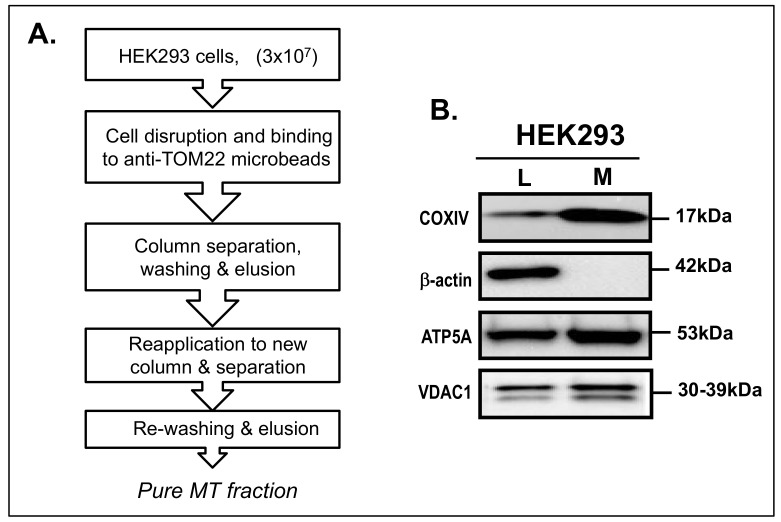
Isolation of pure MT fraction from HEK293 cells. (**A**). A modified protocol for MT fractionation from a HEK293 cell using the Mitochondrial Isolation Kit, human (Miltenyl biotech). The procedure was modified to use more cells (3 × 10^7^), glass homogenization, repeated column separation and extensive washing. (**B**). Evaluation of MT purity by western blotting of MT markers in total cell lysate (L), and MT fractions (M). The antibodies used were anti-COXIV, anti-β-actin, anti-ATP5A and anti-VDAC1. Anti-COXIV and anti-β-actin were used as specific protein markers for MT and cytoplasm, respectively. MT fraction was free from cytoplasmic contamination due to absence of β-actin expression. Antibodies (left) and molecular weights in kDa (right) are indicated.

In order to obtain purely separated MT fraction, the protocol was modified. The number of cultured cells was increased to 3 × 10^7^ and the protocol was optimized as shown in Figure 1A.

In this MT fraction, the signal for COXIV was clearly observed. However, a signal for β-actin was not detected (Figure 1B). These indicated proper isolation of pure MT fraction. To further confirm MT isolation, other markers of MT; ATP5A and VDAC1 (upper band) were also confirmed by western blotting (Figure 1B).

### 3.2. Dp71 Is Detected in MT Fraction of HEK293 Cells

**Figure 2:**
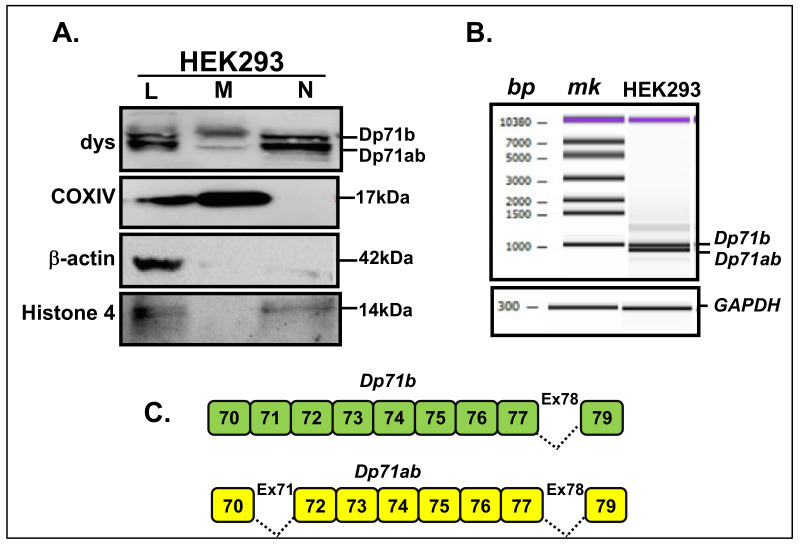
Identification of Dp71 from MT fraction derived from HEK293 cells. (**A**). Western blotting assay of total cell lysate (L), MT fraction (M) and nuclear fractions (N) detected with anti-dys (dystrophin), anti-COXIV, anti-β-actin and anti-Histone 4 (nuclear marker) antibodies. Dystrophin was detected as double bands in MT fraction (M). Antibodies (left) and Dp71 isoforms or molecular weights in kDa (right) are indicated. (**B**). Partial electrophoregram of RT-PCR of *dystrophin* mRNA corresponding to exon 70–79 region in HEK293. The basepair (bp) and marker (mk) locations (above), and bands for Dp71 subisoforms (left) are indicated. *GAPDH* was PCR-amplified as an internal control. (**C**). Exon structures of the two types of Dp71 subisoforms identified: *Dp71b* (without exon 78, green) and *Dp71ab* (without exons 71 and 78, yellow). Shaded boxes and numbers indicate non-deleted exons while dash lines indicate deleted exons.

Subsequently, a western blot assay for dystrophin was conducted. In addition, to confirm our previous observation of dystrophin expression in HEK293, the protein expression pattern from isolated MT (M) was compared to the total lysate (L) and nuclear (N) fractions isolated in parallel. As a result, two bands were revealed in the MT fraction at the same position as those identified in the nuclear fraction (Figure 2A). Comparatively, it was concluded that the upper and lower bands were Dp71b and Dp71ab, respectively, since these bands were determined as Dp71b (without exon 78) and Dp71ab (without both exons 71 and 78) in the nucleus of HEK293 cells [27]. Detection by another dystrophin antibody to the c-terminal, dystrophin (7A10), disclosed a similar finding (Supplementary Figure S1).

To investigate the ability to produce these subisoforms in the cell, *dystrophin* mRNA was analyzed using cDNA prepared from HEK293 cells with primers spanning *DMD* exons 70 to 79. The amplified PCR product was displayed as a double band (Figure 2B). Cloning and sequencing of the PCR product indicated two patterns. The upper and lower bands were *Dp71b* and *Dp71ab*, respectively. The partial exon structure of *Dp71b* and *Dp71ab* isoform is illustrated with the deleted exons indicated (Figure 2C). The pattern of Dp71 subisoforms disclosed in the cDNA reflected the observations in the MT protein expression (Figure 2A).

### 3.3. Dp71 Expression in MT Fraction of Other Human Cancer Cells

**Figure 3:**
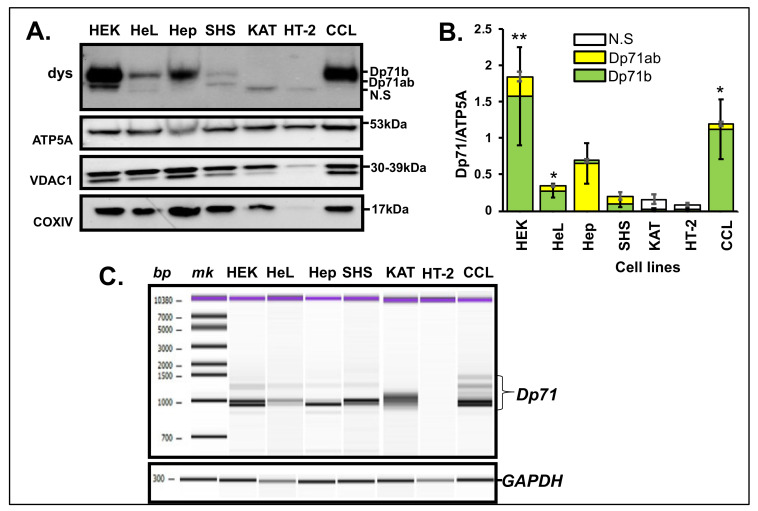
Identification of dystrophin from MT fraction from other cancer cells. (**A**). Western blotting of dystrophin from MT fractions from HeL (HeLa), Hep (HepG2), SHS (SH-SY5Y), KAT (KATOIII), HT-2 (HT-29) and CCL (CCL-136) detected with anti-dys (dystrophin), anti-ATP5A, anti-VDAC1 and anti-COXIV antibodies. HEK (HEK293) was included for comparison. Various Dp71 expression patterns were displayed. Antibodies (left) and Dp71 isoforms or molecular weights in kDa (right) are indicated. (**B**). The ratio of the density of Dp71 subisoforms to ATP5A displayed as a stacked column plot comparing the abundance of Dp71b and Dp71ab. The results showed statistically significant abundance of Dp71b in the MT fractions of HEK293 (** *p* < 0.01), HeLa (* *p* < 0.05) and CCL-136 (* *p* < 0.05). Green and yellow bars indicate Dp71 and Dp71ab, respectively. N.S, non-specific. (**C**). Partial electrophoregram of RT-PCR of *dystrophin* mRNA corresponding to exon 70–79 region in cell-derived cDNAs. The basepair (bp) and marker (mk) locations (above), and bands for Dp71 subisoforms (left) are indicated. *GAPDH* was PCR-amplified as an internal control.

The MT localization of Dp71 subisoforms in HEK293 cells prompted the investigation of MT dystrophin from other cultured cells. Consequently, MT fractions were extracted from HeLa cervical carcinoma cells, HepG2 hepatic carcinoma cells, SH-SY5Y neuroblastoma cells, KATOIII gastric carcinoma cells, HT-29 colon carcinoma cells and CCL-136 rhabdomyosarcoma cells. MT fractions were confirmed by positive MT-specific expression of COXIV and negative β-actin expression (data not shown). The MT fractions were assayed by western blotting for dystrophin isoforms (Figure 3A). By comparison of the Dp71 expression pattern in HEK293, HepG2 and CCL-136, cells displayed a single larger dystrophin product while HeLa and SH-SY5Y cells exhibited two dystrophin bands each. Remarkably, KATOIII and HT-29 cells revealed a single smaller size band.

We supposed that the upper band and lower bands could be Dp71b and Dp71ab, respectively. Therefore, the relative abundance of Dp71 subisoform in each cell line was determined from the ratio of each Dp71 isoform to ATP5A (Figure 3B). The ratio differed from cell to cell with CCL-136 presenting the highest ratio after HEK293. This result was statistically significant (*p* < 0.05), signifying high abundance of Dp71b compared to Dp71ab in MT fraction of CCL-136 as well (Figure 3B).

To demonstrate the ability of each cell to produce these isoforms, a fragment extending from *DMD* exon 70 to 79 was RT-PCR amplified in the respective cell-derived cDNA (Figure 3C). The band densities of the PCR products seemed to correspond to the findings of western blotting. However, to further confirm the genomic content, each of the PCR products was purified and sequenced. Results from sequencing clarified that of RT-PCR and reflected the result of western blotting. From HeLa cells, a large transcript encoding *Dp71b* was amplified as a major product. This matched well with Dp71b identification in MT fraction by the western blotting analysis. In addition, HepG2 cells produced a smaller size product as a major product that corresponded to *Dp71ab*. Therefore, the single western band from MT analysis was decided as Dp71ab. Moreover, from SH-SY5Y cells, two bands corresponding to *Dp71b* and *Dp71ab* were revealed, with *Dp71b* being the predominant product. These products matched those disclosed in MT fraction.

From KATOIII cells, the product obtained was not clear and from HT-29 cells, no band was amplified. Hence, the bands observed from the MT fraction derived from these cells were determined as artifactual or non-specific (N.S; Figure 3A). Similar to HEK293, two types of *Dp71* subisoform transcripts were mainly identified here, namely *Dp71b* and *Dp71ab*.

### 3.4. Dp71 Expression in MT Fraction from Primary Cultured Myocytes

Myocytes were established from two DMD patients (DMD1 and DMD2) and one Becker muscular dystrophy (BMD; a milder form of DMD) patient (DMD3). The clinical characteristic of each patient is described in Table 1. All patients presented with a point mutation in *DMD* exon 19, 21 or 31, respectively. The point mutation led to a nonsense mutation in the *DMD* gene in each case. While DMD1 and DMD3 are still ambulant, DMD2 lost ambulation at 12 years of age (Table 1).

**Figure 4:**
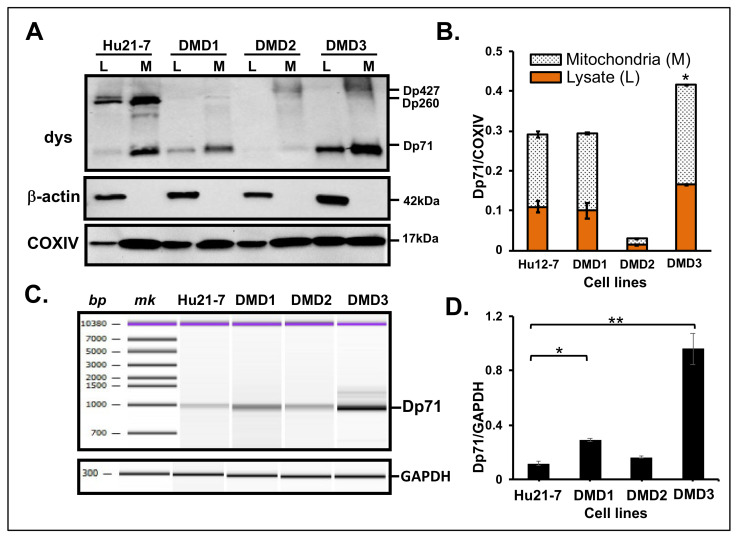
Identification of dystrophin from MT fraction extracted from primary cultured myocytes from dystrophinopathy patients (DMD1, DMD2 and DMD3). (**A**). Western blotting of dystrophin from total lysate (L) and MT fractions (M) of DMD1, DMD2 and DMD3 with ant-dys (dystrophin), anti-β-actin and anti-COXIV. Normal skeletal muscle Hu-12 was included for comparison. Antibodies (left) and Dp71 isoforms or molecular weights in kDa (right) are indicated. (**B**). The ratio of the band density of Dp71 isoform to COXIV displayed as a stacked column plot comparing Dp71 expression in total lysate (L, orange bar) and MT fraction (M, patterned bar). The data showed statistically significant abundance of Dp71b in the MT fractions of DMD3 (* *p* < 0.05). Black and dotted bars indicate lysate and MT fraction, respectively. (**C**). Partial electrophoregram of RT-PCR of *dystrophin* mRNA corresponding to exon 70–79 region in cell-derived cDNAs. The basepair (bp) and marker (mk) locations (above), and bands for Dp71 isoform (left) are indicated. *GAPDH* was PCR-amplified as an internal control. (**D**). The ratio of the peak height of Dp71 isoform to GAPDH showed statistically significant abundance of Dp71 in the cDNAs of DMD1 (* *p* < 0.05) and DMD3 (** *p* < 0.01).

MT dystrophin expression in these myocytes and the normal skeletal muscle control (Hu21-7) was evaluated. The results of western blotting were assessed in the total lysate (L) and MT fraction (M) derived from each myocyte (Figure 4A). From the results, dystrophin was revealed as a 427 kDa band in the normal control lysate but not in DMD/BMD myocyte-derived lysates (Figure 4A). MT fraction isolated from these cells displayed signals for dystrophin as Dp71 in all samples. Amongst the DMD/BMD myocytes, abundant expression of Dp71 was visualized in DMD3 myocytes followed by DMD1 and lastly, DMD2, respectively (Figure 4A).

The amount of dystrophin was higher in the MT fractions of both the control and DMD/BMD samples when compared to the respective total lysate for each cell. For unbiased quantification, the Dp71 expression in total lysate and MT was normalized with COXIV (Figure 4B). The Dp71 expression level was clearly seen to be significantly higher in the MT fraction of DMD3 patients’ samples compared to Hu21-7. This result was statistically significant (*p* < 0.05) (Figure 4B).

Furthermore, the *Dp71* transcript was examined in the patient’s myocyte-derived cDNA. Amplification of the region encompassing *DMD* exons 70 to 79 revealed a similar pattern in all samples with varying densities. Consistent with the findings of western blotting, this band was determined as *Dp71* transcript (Figure 4C). Remarkably, after normalization with *GAPDH*, the amount of *Dp71* transcript obtained by cDNA analysis matched that of the western blotting with DMD3 > DMD1 > DMD2 (Figure 4D).

### 3.5. Interaction of Dystrophin with ATP5A

**Figure 5:**
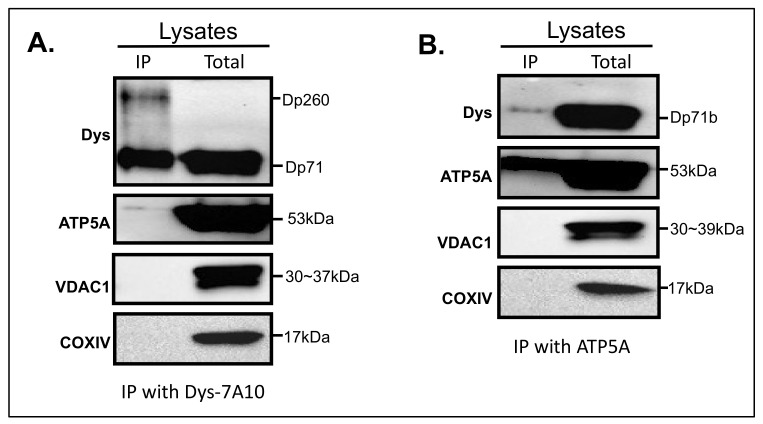
Immunoprecipitation assay (**A**). Immunoprecipitants of dystrophin were purified using mouse anti-dystrophin (7A10): sc-47760 monoclonal antibody conjugated Dynabeads^®^ M-270 Epoxy and separated by SDS-PAGE and western blotting with antibodies recognizing dystrophin, ATP5A, VDAC1 and COXIV proteins. Antibodies (left) and Dp71 isoforms or molecular weights in kDa (right) are indicated. Only anti-ATP5A showed slight reactivity to immunoprecipitated of dystrophin. (**B**). Immunoprecipitants of ATP5A were purified using mouse anti-ATP5A: ab14748 monoclonal antibody conjugated Dynabeads^®^ M-270 Epoxy beads and separated with SDS-PAGE and western blotting with antibodies recognizing dystrophin, ATP5A, VDAC1 and COXIV proteins. Only anti-dys showed slight reactivity to immunoprecipitants of ATP5A. Antibodies (left) and Dp71 isoforms or molecular weights kDa (right) are indicated.

To further characterize Dp71 in MT fraction, Co-IP indicated that dystrophin was well precipitated as Dp71 (Figure 5A). Remarkably, ATP5A was slightly immuno-reactive to dystrophin. Results with the normal rabbit IgG indicated that no non-specific binding was detected (Supplementary Figure S2). Other MT markers were not reactive to dystrophin (Figure 5A).

A reciprocal experiment with the ATP5A antibody indicated that dystrophin was disclosed as a single band corresponding to Dp71 (Figure 5B). This band was identified with a similar intensity to the precipitated ATP5A in Figure 5A. ATP5A was well precipitated in this assay, as expected. However, other MT markers, VDAC1 and COXIV, were not precipitated together with Dp71 (Figure 5B).

From these results, it was supposed that the dystrophin antibody might be able to co-immunoprecipitate ATP5A and this effect was mutually displayed in a reciprocal assay with ATP5A.

## 4. Discussion

In this report, Dp71, the ubiquitous isoform of dystrophin, was first shown to localize to the MT fraction of human cells through the employment of a super-magnetic bead-based strategy to isolate pure MT from human cells. This strategy uses magnetic microbeads conjugated to the antibody, TOM22, a human MT outer membrane protein [32]. The quality, enrichment, and purity of isolated MT has been shown as being comparable to MT obtained using the ultracentrifuge method [32]. The MACS method has been utilized as the method to isolate MT in many studies [36,37,38]. However, the isolated MT fraction by the initial protocol showed a contamination that was disclosed through the detection of signals for β-actin (data not shown). Such impurity has been observed in the study of lipid profiling of MT from human cultured cells [39]. Our results were compatible with this, although we modified the protocol to use more cells, complete cell disruption with a glass homogenizer and add more washing steps (Figure 1A). By this modified method, the isolated MT fraction was rendered free of any signals for β-actin. However, signals for MT inner and outer membrane proteins were positive (Figure 1B). We concluded we had obtained highly purified MT fraction. In this fraction, dystrophin was revealed as two bands with a rather different pattern to the subisoforms from total lysate. This finding further supported the efficient isolation of MT.

It is possible that with the current increase of the number of proteins localized to MT, especially proteins not directly linked with ATP production, there is a high probability for dystrophin to be expressed in MT from two points of view: (1) the pathology of DMD is well explained by MT dysfunction [40,41], (2) MT functional aberration has been postulated to be a primary rather than secondary event in DMD [42]. Our finding is therefore expected to pave the way for elucidation of dystrophin deficiency-induced MT dysfunction. Such studies seem promising for the identification of new targets for DMD treatment that ameliorate MT dysfunction in DMD.

To the best of our knowledge, only one report studying dystrophin in MT has been published. Chávez et al., (2000) analyzed dystrophin in MT of *mdx* mouse brain [26]. Our finding is the first to show dystrophin expression in MT in human cells, and dystrophin in MT was mostly Dp71b and Dp71ab. Accordingly, we have previously reported that all *dystrophin* transcripts in HEK293 cells lacked exon 78 [27]. Therefore, the identification of Dp71 in human MT is perhaps acceptable. Chávez’s group also identified Dp71f in MT of mouse kidney and bovine heart [26]. This finding strongly suggested the relationship of dystrophin and MT.

Dp71 subisoform production was cell line-specific, and the ability to produce Dp71 irrespective of cells was demonstrated by the presence of mRNA (Figure 3A). The absolute quantification of the Dp71 mRNA expression in the cell-derived cDNA will provide more information of the Dp71 mRNA expression profiles. In recent studies, dystrophin (Dp427) has been implicated in tumorigenesis of soft tissue tumors and therefore coined a “tumor suppressor” and a likely anti-metastatic agent [43]. In addition, Dp71 isoform was suggested to be essential in these tumors [44,45]. Therefore, it was expected that SH-SY5Y and CCL-136, both originating from skeletal muscle progenitor cells, will exhibit higher expressions of Dp71 subisoforms (Figure 3B). However, this was not the case for SH-SY5Y, supporting the complex heterogeneity of the dystrophin protein expression. DMD patients have been observed to be complicated by brain tumors such as medulloblastoma [46,47] and glioma [48]. However, the mechanism has not been clarified. It will be beneficial to evaluate the MT Dp71 in such cases.

Interestingly, the density of Dp71 varied among the MT fraction derived from primary cultured myocytes. The lower level of Dp71 may suggest MT impairment in DMD, whereas higher levels of Dp71 might indicate less severe phenotypes in the BMD derived myocytes. In fact, DMD3 carry a nonsense mutation in the *DMD* gene which does not lead to alteration of the reading frame of the mRNA. Hence, a biopsied muscle from the patient exhibited patches of staining of dystrophin comparable to a BMD patient [49]. MT myopathy has been suggested as a primary effect to explain the pathophysiology of DMD [23,24]. From this hypothesis, the low Dp71 expression in DMD derived myocytes could be induced by high protein turnover [50]. Mitochondria in dystrophic myofibers have been shown to respond poorly to sarcolemma injury. These mitochondrial deficits also reduce the ability of dystrophic muscle cell membranes to repair and regenerate. It is therefore plausible that the state of mitochondrial impairment could be related to the clinical severity. Hence, the importance of elucidating the MT dystrophin among DMD and BMD patients [40]. Although the expression of MT Dp71 seems to be correlated to the clinical severity in this study, more patients’ myocytes will be needed to obtain a definitive answer.

Dp71 has been reported to be absent in skeletal muscles [13], nonetheless, one study recently detected Dp71 in the skeletal muscle of human, normal and *mdx* mouse by an automated capillary western assay system. They also showed that Dp71 was expressed in HEK293 cells overexpressing Dp71 and skeletal muscle progenitor cells, and in CRL-2061 rhabdomyosarcoma cells. In addition, they reported that human skeletal muscle samples varied widely in the expression of Dp71 [51]. However, they did not investigate on the subcellular localization of the Dp71 isoform in these cells. The present study confirmed the expression of Dp71 in human skeletal muscle cells and similarly to the findings of Kawaguchi and coworkers [51], the expression of Dp71 varied among samples and also MT fractions from primary myocytes. The significance of the difference in the expression levels remains to be elucidated.

In DMD, Dp71 has been implicated in many neuro-psychiatric diseases. Mental retardation (MR) had been reported in about 30% of DMD patients and the severity of MR may account for a shift of 2 SD downward of the intelligence quotient [52]. However, the exact mechanism by which the Dp71 abnormality impacts MR is still unknown. In addition, MT dysfunction has been suspected to be involved in intellectual disability-related diseases [53] while autism complicates DMD in about 21% of patients [54]. Even in autism, MT dysfunction was expected to be connected to disease etiology [55]. Considering that Dp71 is the most abundant Dp isoform expressed in the brain [13], it is possible that these neuro-psychiatric complications would in the future be explained from the standpoint of a specific MT Dp71 isoform.

Short stature has been described in DMD [56,57,58]. However, the mechanism of this is still unknown. One study revealed a high incidence of short stature in a sub-group of 18 DMD patients with the *Dp71* mutation [58]. They reported that in DMD, higher incidence of short stature (height SDS  < −2.5 SD) was observed in the *Dp71* subgroup that had mutations in *DMD* exons 63–79, indicating that Dp71 might have a role in growth regulation [58]. Therefore, MT dysfunction triggered by Dp71 abnormality could be implicated in short stature in DMD.

For a better understanding of the implication of MT Dp71 on DMD and MT-related diseases, interactive proteins to Dp71 in the MT will be warranted. Although we identified a possible interaction between dystrophin Dp71 and ATP5A while employing DMD3 myocytes, only one sample type was used, and the absence of a known positive control makes this a limitation in this study. More data is required for clarification.

Several useful databases for mitochondrial protein information [59,60], as well as MT proteome-wide studies [61] are available to evaluate already identified totally- and subcellularly-localized MT proteins. By assessment of MT localization of dystrophin or Dp71 globally through MitoMiner 4.0, a database of mammalian mitochondrial localization evidence [62], three entries were found that suggested MT localization of dystrophin with no information on the isoform type, and one entry for “Dp71” was also present. One entry each of dystrophin and Dp71 was retrieved from analysis of human skeletal muscle-derived MT proteome using one-dimensional gel electrophoresis and HPLC ESI MS/MS [63], whereas the other two entries were supported by experimental evidence from Mass spectrometry (MS) using mouse tissue [64]. No further functional investigation was conducted in all three entries for confirmation. These results suggest that dystrophin might be localized in the mitochondria, and our present study might just have added to the findings. Inconsistency of protein expression from MS analysis of the mitochondria proteome might be due to tissue differences [61], or that the expression might be very low.

The MT is an important subcellular organelle for energy production through respiration, and hence its defect is implicated in many human diseases [65]. Many studies aimed at amelioration of MT function through drug delivery therapy to the MT [66] in cancers [67], DMD [68] and other MT-related diseases are ongoing [66]. In dystrophin-deficient skeletal muscle, various MT-implicated metabolic defects have been reported. Glycolysis [69], fatty acid metabolism [70] and MT electron transport chains [71] have been evaluated to understand DMD pathology, leading to a wealth of knowledge in the relationship between them. However, it is now recommended that MT function in DMD be analyzed from the starting point of dystrophin expression in MT. Investigation of the dystrophin expression in MT could inform research into new MT-targeted therapies in dystrophin-related diseases, thereby implicating MT aberrations.

## 5. Conclusions

We described here the identification of Dp71 subisoforms in MT fractions from human cancer cells. The observed Dp71 subisoforms were cell-specific with most cells expressing Dp71b and Dp71ab. MT fractions from primary cultured myocytes from two DMD and one BMD patients, all with a nonsense mutation in the *DMD* gene, demonstrated only Dp71 expression with varying densities. MT Dp71 was higher in BMD patient-derived myocytes. It is anticipated that studies to elucidate MT dysfunction in DMD will be evaluated from the standpoint of MT dystrophin expression.

## Figures and Tables

**Table 1:** Characteristics of dystrophinopathy patients.

	DMD1	DMD2	DMD3
Mutation	c.2308A > T (Exon 19) p.Lys770 *	c.2677C >T (Exon 21) p.Gln893 *	c.4303G >T (Exon 31) p.Glu1436 *
Phenotype	DMD	DMD	BMD
Symptoms	Calf pseudohypertrophy (8yo) Waddling gait (<8yo) Climb stairs with support (<8yo) Gowers’s sign (8yo)	Calf pseudohypertrophy (3yo) Waddling gait (6yo) Climb stairs with support (7yo)	None related to muscle weakness (2yo)
Muscle biopsy	No staining with DYS1-3 ab (8yo)	No staining with DYS1-3 ab (8 yo)	Patchy and faint staining with DYS1-3 ab (4yo)
Present situation	11yo; Ambulant	15yo; Non ambulant	14yo; Runs normally, riding daily to school

yo; year old, DYS1-3 ab; denotes DYS1, DYS2 and DYS3 dystrophin antibodies against the Rod, C-terminal and N-terminal domains, respectively. * indicates presence of a stop codon and hence a nonsense mutation

## Data Availability

The data presented in this study are available on request from the corresponding author.

## Supplementary Materials

The following are available online at https://www.mdpi.com/article/10.3390/life11090978/s1, Figure S1: Detection of Dp71 by another dystrophin C-terminal antibody, Figure S2: Immunoprecipitation assay.
