# Supplementary material for: Dystrophin Dp71 Subisoforms Localize to the Mitochondria of Human Cells
Source: Life (Basel). 2021 Sep 16;11(9):978. doi: 10.3390/life11090978 (PMC8468555; doi:10.3390/life11090978)
Supplement: Supplementary file 1 [file life-11-00978-s001.zip › life-1287398-supplementary.pdf]

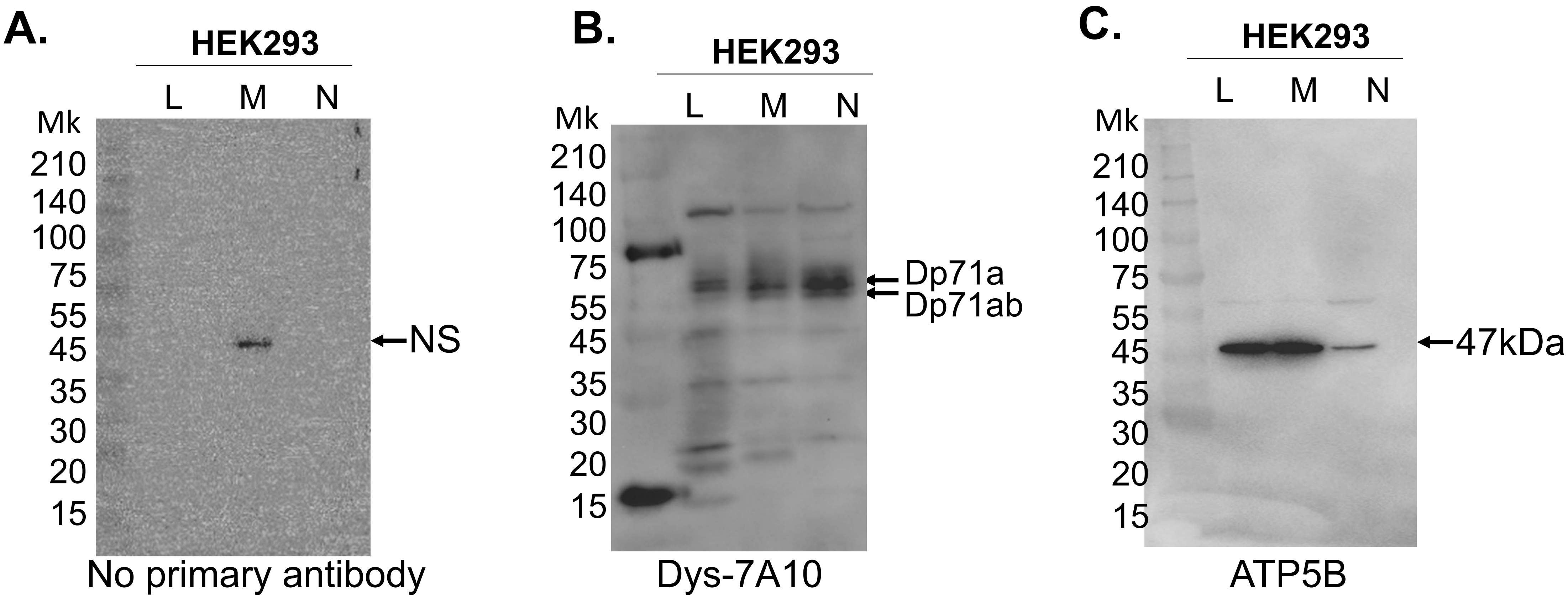

Figure S1: Detection of Dp71 by another dystrophin C-terminal antibody by western blotting in total cell lysate (L), MT fraction (M) and Nuclear fraction (N) of HEK293 cells. **A**. "No primary antibody" control to check for nonspecific bands that could be produced by secondary antibody, anti-mouse HRP. **B.** Detection of Dp71 by anti-mouse Dys-7A10 (1:500; sc47760). **C.** Anti-goat ATP5B (1:200; Sc-16690) was used as MT marker. Antibodies (bottom) and Dp isoforms or molecular weights in kDa (right) are indicated. NS indicates nonspecific. Mk, marker (Pre-stained XL-ladder; Invitrogen, Thermofisher Scientific Inc, MA, USA).

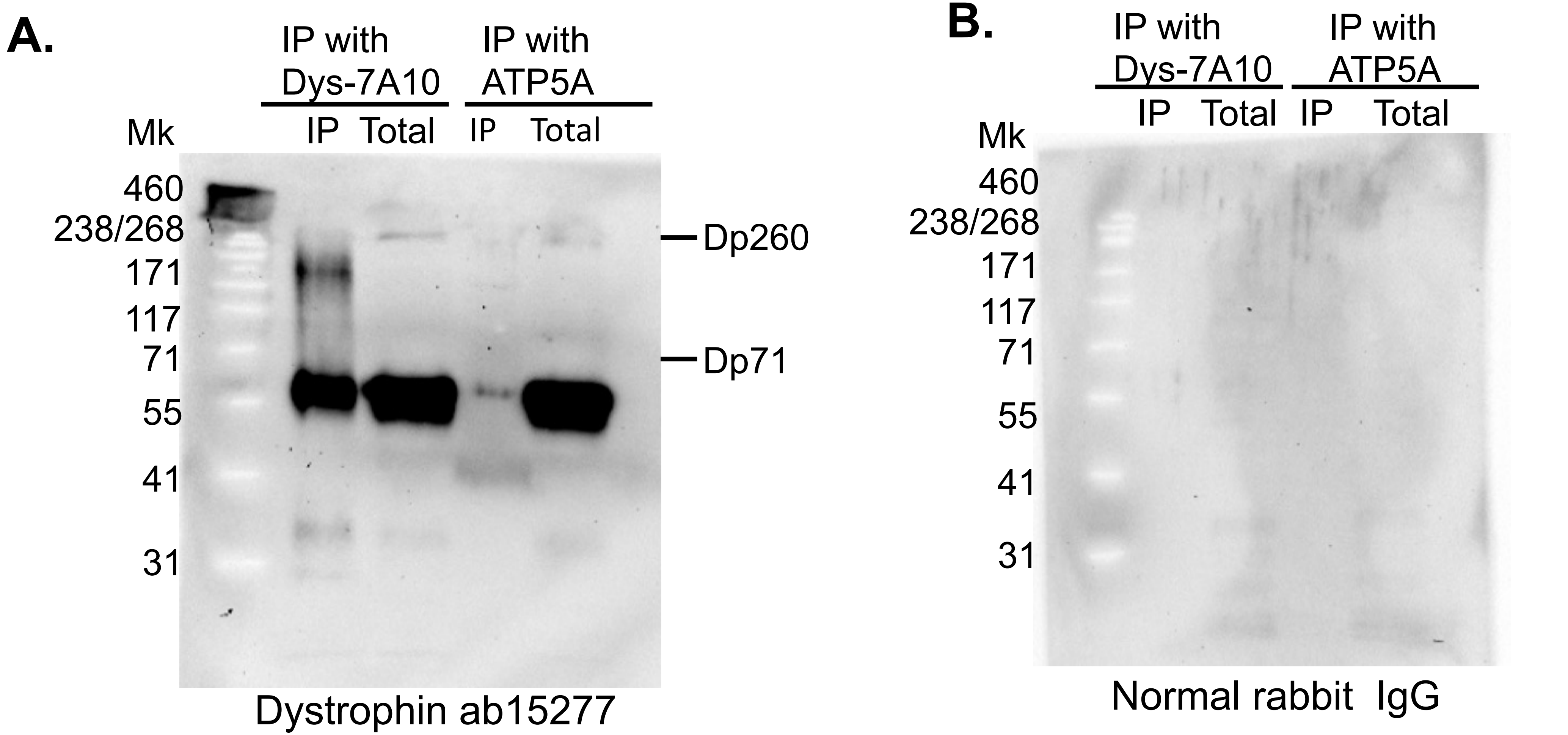

Figure S2: Immunoprecipitation assay: **A**. Immunoprecipitants of Dys-7A10 and ATP5A detected with anti-rabbit dystrophin ab15277 (1:1000). **B**. Immunoprecipitants of Dys-7A10 and ATP5A detected with normal rabbit IgG (1:1000). Antibodies (bottom) and Dp isoforms or molecular weights in kDa (right) are indicated. Mk, marker (HiMark Pre-Stained Protein Standard; Invitrogen, Thermofisher Scientific Inc, MA, USA).
